# Mitochondrial Stress Induces Plant Resistance Through Chromatin Changes

**DOI:** 10.3389/fpls.2021.704964

**Published:** 2021-09-22

**Authors:** Ana López Sánchez, Sofía Hernández Luelmo, Yovanny Izquierdo, Bran López, Tomás Cascón, Carmen Castresana

**Affiliations:** Genética Molecular de Plantas, Centro Nacional de Biotecnología, Madrid, Spain

**Keywords:** mitochondrial stress, arabidopsis, induced resistance, plant stress, epigenetics, plant defense, chromatin modifications, priming

## Abstract

Plants respond more efficiently when confronted with previous similar stress. In the case of pathogens, this memory of a previous infection confers resistance to future ones, which possesses a high potential for agricultural purposes. Some of the defense elements involved in this resistance phenotype, as well as epigenetic mechanisms participating in the maintenance of the memory, are currently known. However, the intracellular cascade from pathogen perception until the establishment of the epigenetic memory is still unexplored. Here, through the induction of mitochondrial stress by exogenous applications of Antimycin A in *Arabidopsis thaliana* plants, we discovered and characterized a role of mitochondrial stress in plant-induced resistance. Mitochondrial stress-induced resistance (MS-IR) is effective locally, systemically, within generation and transgenerationally. Mechanistically, MS-IR seems to be mediated by priming of defense gene transcription caused by epigenetic changes. On one hand, we observed an increment in the deposition of H3K4me3 (a positive epigenetic mark) at the promoter region of the primed genes, and, on the other hand, the DNA (de)methylation machinery seems to be required for the transmission of MS-IR to the following generations. Finally, we observed that MS-IR is broad spectrum, restricting the colonization by pathogens from different kingdoms and lifestyles. Altogether, this evidence positions mitochondria as a prominent organelle in environment sensing, acting as an integrating platform to process external and internal signals, triggering the appropriate response, and inducing the epigenetic memory of the stress to better react against future stressful conditions.

## Introduction

Along the 500 million years inhabiting the surface of the earth, plants have developed such phenotypic plasticity that even when being sessile, they can adapt and complete their biological cycle in adverse environmental conditions (Wilkinson et al., 2019). Environmental changes are called “stress” when they importantly impact the plant growth, development, and, depending on the intensity and length, put at risk the plant viability (Gaspar et al., 2002; Kranner et al., 2010). Such stressful conditions are the root cause of agricultural losses and the reduction of crop yields (Bailey-Serres et al., 2019). Accordingly, increasing plant resistance to stress has been one of the major objectives of plant breeders and biotechnologists. In the case of biotic stresses (virus, bacteria, fungus, herbivores, etc.), plants count on constitutive barriers, such as the plant cell wall or trichomes, but also, on a complex, effective, and inducible immune system, which operates specifically when the plant is challenged by a pathogen (Jones and Dangl, 2006). The stress perception typically leads to an increment in Ca^++^ levels, a burst in reactive oxygen species (ROS) and a phosphorylation cascade mediated by MAPKs. Afterwards, a reprogramming in gene expression which ends in physiological and metabolic changes, aims to stop the plant growth and focuses resources on the restriction of the pathogen colonization and endurance of the period of disease (Nürnberger and Scheel, 2001). Interestingly, these effective elements could not fully explain the plant phenotypic plasticity observed in nature, supporting the existence of a complementary layer of regulation for the innate immune system. Probably, due to the recurrent character of some stresses in nature, plants can respond more effectively when they have been previously exposed to similar experiences. This effect, which involves certain memory of the stress and controls different factors of the innate immune system, is known as induced resistance (IR) and manifests as a reduced susceptibility to following pathogen attacks (Sequeira, 1983; Prime-A-Plant Group et al., 2006; Hilker et al., 2016; De Kesel et al., 2021).

There have been many different types of IR described to date. In some cases, after a first local transmission, the defense signal can reach distal parts of the plant triggering IR systemically and protecting the entire plant, including tissues that were not directly exposed to the stress. The progressive understanding of IR-triggering stimuli has promoted the identification of different IR agents, including pathogen infections, pathogen-indicating signals, defense-related hormones, or even exogenously applied compounds, such as β-aminobutyric acid (BABA). The durability of IR is another aspect that has raised great interest. Depending on the stability of the stress memory, IR phenotypes can last for short periods (few days), longer periods (weeks, months, or years, depending on the species) or even to be transmitted to the next generations of plants, which is known as “transgenerational-induced resistance” (*t*-IR, Luna et al., 2012; Mauch-Mani et al., 2017). Regarding the molecular mechanisms involved, the IR phenotypes are currently associated with two main non-exclusive mechanisms, whose contribution seems to depend on the nature of the interaction as well as the life strategy of the plant species: direct induction of defenses and immune priming (Wilkinson et al., 2019). Priming is characterized by a more effective induction of innate immunity (usually faster and/or stronger) in subsequent pathogenic interactions. Numerous studies have corroborated the relationship between epigenetics and priming processes at different levels (López et al., 2011; Luna and Ton, 2012; López Sánchez et al., 2016; Lämke and Bäurle, 2017). The main epigenetic mechanisms are related to the control of chromatin structure. These include the methylation of cytosine residues of the DNA, chemical modification of histone proteins, deposition of histone variants, and the general positioning of nucleosomes (Roberts and López Sánchez, 2019). The modification of those epigenetic marks produces changes in gene inducibility that can be stable during long periods of time, or even heritable. Such epigenetic changes have been detected in response to infection, other IR agents (Jaskiewicz et al., 2011; Dowen et al., 2012), and in t-IR processes (Luna et al., 2012; Stassen et al., 2018); whereas mutant plants defective in chromatin modifications show priming phenotypes (López et al., 2011). Regarding this evidence, the epigenetic mechanisms have become the most probable molecular machinery mediating priming processes and memory of stress (Lämke and Bäurle, 2017).

Albeit the epigenetic machinery has been extensively demonstrated to play a role in plant defense and priming processes, the intracellular cascade going from pathogen perception to the epigenetic changes mediating IR is still unknown. In this respect, mitochondria seem to process external and internal signals to properly coordinate plant defense responses. In this integration, molecules, such as the defense-related hormone salicylic acid (SA), nitric oxide (NO), and reactive oxygen species (ROS), play a key role with a direct and/or indirect effect in mitochondria (Colombatti et al., 2014). The mitochondrion is the cellular compartment where aerobic respiration takes place (Taylor, 2018). In order to obtain energy, it oxidizes organic-reduced compounds, activating electron transitions through the electron transport chain (ETC), producing CO_2_, H_2_O, and free energy in the form of ATP (Schertl and Braun, 2014). Additionally, it is also the place where the regulation and the interaction of many different synthetic pathways occur, that is, the case of general intermediates for the biosynthesis of nucleic acids, amino acids, and fatty acids, as well as specific ones participating in glycine metabolism, folic acid, and ascorbic acid synthesis (Day, 2004). The role of mitochondria in plant-pathogen interactions has been previously addressed (Amirsadeghi et al., 2007; Sasan et al., 2007; Colombatti et al., 2014). Following pathogen recognition, there is an increment in the production of 9-Lipoxygenase-derived oxylipins, which signal for defense in a process dependent on specific mitochondrial proteins and associate processes (Vellosillo et al., 2013; Izquierdo et al., 2018). Moreover, the pathogen perception causes a dysfunction of the mitochondrial ETC, which increases the production of ROS (Amirsadeghi et al., 2007). This is necessary to trigger the expression of the defense genes and the programmed cell death involved in locally confining the pathogen and promoting the plant systemic signals involved in IR. As part of the defense response to pathogens, the hormone SA is produced. Its concentration levels can be perceived at the mitochondria by its binding to the complex II of the ETC (Belt et al., 2017), exerting positive feedback of the system (increasing ROS). However, the specific role of mitochondria in different IR phenotypes has not been studied. Within this framework, mitochondria do not appear to be fully explored candidates to play a role in the intracellular signaling of IR.

Here, we addressed the role of mitochondrial stress in IR by the exogenous application of the ETC inhibitor, Antimycin A (AA), in *Arabidopsis* plants. AA disturbs the ETC at the level of the complex III, promoting mitochondrial stress and increasing ROS and NO levels. We observed that mitochondrial stress induces a strong pathogen resistance phenotype (MS-IR). This MS-IR requires an intact mitochondrial-nucleus retrograde signaling, and it is independent of SA synthesis, but NPR1 dependent. We discovered that MS-IR is effective locally, systemically, and long-lasting, conferring protection even to the next generations of plants. Our results indicate that MS-IR is mainly achieved by priming at the level of gene expression rather than the direct induction of defenses. In addition, this primed state triggered by mitochondrial stress seems to be mediated by epigenetic mechanisms. On the one hand, it is associated with an open chromatin conformation due to an increased deposition of the positive epigenetic mark H3K4me3 at the promoter regions of some defense-related genes; and, on the other hand, the transgenerational transmission of MS-IR is DNA (de)methylation dependent. Lastly, to estimate the specificity of the MS-IR, we tested its effectiveness against pathogens from different kingdoms and lifestyles. We observed resistance against all pathogens analyzed, indicating that mitochondrial stress confers broad spectrum resistance. In summary, our study identified mitochondrial stress as an IR stimulus and positioned mitochondria as a plausible cellular integrating platform, processing the external information and establishing the memory of the stress through specific epigenetic changes.

## Materials and Methods

### Plant Material and Growth Conditions

All *Arabidopsis thaliana* lines described in this study are in the genetic background of accession Col-0 (NCBI, Tax ID 3702). Lines *AOX1a promoter:GUS* and *ANAC017 OE* (Van Aken et al., 2016) were kindly provided by Prof. J. Whelan and Dr. O. Van Aken, Ws-*NahG* by Dr. J. A. Ryals, *sid2* by Prof. J. P. Métraux, and *npr1-1* by Prof. X. Dong. *nrpe1-11* (SALK_029919) and *ros1-4* (SALK_135293) mutant seeds were gently gifted by J. Ton's laboratory and previously reported in López Sánchez et al. (2016). All plants were stratified 3 days in darkness at 4°C, sown in *Jiffy-7* peat pellets, and grown in short-day conditions (9-h light at 21°C and 15-h night at 19°C) at 60% relative humidity (RH) and 125 μmol/s.m^2^light intensity. Growing plants were watered periodically each 3 days by flooding the trays for 0.5 h and removing the excess of water afterwards. In experiments where sample collection was needed (RNA and ChIP analysis), randomized block design was used. Each block consisted of one to two trays containing a representative number of the different treatments. Each 3 days, the trays were rotated inside the block, and the block was rotated to a different position in the growth cabinet. The samples were collected from individuals in the same block, constituting each block a biological replicate. For phenotyping analysis, the different lines/treatments were equally distributed in the different trays, and the trays were rotated each day, for 3 days, to different positions in the growth cabinet. All the experiments were repeated at least three times with similar results.

### AA Treatments

Antimycin A (AA, Sigma A-8674; Sigma-Aldrich Corp., St. Louis, MO, USA) at 50 μM in 0.02% Tween 20 (Sigma P-1379; Sigma-Aldrich Corp., St. Louis, MO, USA) was applied by spraying similarly to the method described in Zarkovic et al. (2005) in the main part of the experiments (MS-IR experiments, gene expression, and ChIP analysis). As AA stock solution is prepared in ethanol, the same volume of absolute ethanol +0.02% tween 20 was applied in the control (Mock) treatments. For local *vs*. systemic IR, plants were treated with AA at 50 μM/Mock by syringe infiltration. For the generation of the AA t-IR plant lines, AA was applied by spraying two times per week for 3 weeks to a total of six treatments (leaving 3–4 days for recovering between treatments). After treatments were finished, the plants were moved to long-day conditions (16-h light/8-h night at 21°C) to trigger flowering and set seeds.

### Quantification of AA-IR

For all within-generation IR experiments, inoculations were performed 3 days after treatment (dat, with AA/Mock). All phenotyping experiments were repeated at least three independent times with similar results.

*Hyaloperonospora arabidopsidis* (*Hpa*). The isolated WAC09 was kindly gifted by the laboratory of Prof. J. Ton. Spores from frozen stocks (−80°C) were maintained in a running culture on Ws *NahG* seedlings, replicated weekly. The inoculum was prepared at 10^5^ conidiospores/ml, from infected plants at 6 days post inoculation (dpi), as described in López Sánchez et al. (2016). In all *Hpa* analysis, 2.5-week-old seedlings (or 4.5-week-old plants in the case of local *vs*. systemic IR analysis) were inoculated by spraying and maintained at 100% humidity. Five dpi, plants (or leaves) were collected in ethanol 96% and trypan blue stained (López Sánchez et al., 2016). Differences in *Hpa* growth were quantified by the visualization of infected leaves with a stereo microscope and its classification to one of the four different *Hpa* colonization classes (I: no visible colonization, II: oomycete growth without sporulation, III: visualization of sporangiophores and asexual sporulation, IV: sexual sporulation).

*Pseudomonas syringae* pv. *tomato* DC3000 (*Pst*). *Pst* was cultivated from a glycerol stock (−80°C) for 48 h on King's B medium (KB) agar plates supplemented with 50 μg/ml rifampicin (Sigma-Aldrich, R3501). Cells were collected, resuspended in 10-mM MgSO_4_ and adjusted by optical density, measured spectrophotometrically (OD_600nm_). The inoculum was prepared at 5 × 10^7^ colony-forming units (CFU)/ml, supplemented with 0.015% Silwet L-77 (Lehle SeedsVIS-02) and applied by spraying. *Pst* growth was assessed at 3 dpi. Using a cork borer (0.75-cm diameter) four leaf discs/infected plants were sampled and placed in 1.5-ml tubes, containing 600-μl 10-mM MgSO_4_. Leaf discs were homogenized in the tubes, using 2-mm glass beads and a cold grinder for 30 s two times, leaving 1-min recovery in between. The homogenized samples were then serial diluted in 10-mM MgSO_4_, using 96-wells microtiter plates (Thermo Scientific^TM^ Nunc^TM^ 96-Well Polystyrene Conical Bottom MicroWell^TM^ Plates, 249570). Twelve samples in each plate were serial-diluted eight times (five-fold dilutions) and plated onto selective KB agar plates, containing 50-mg/ml Rifampicin (Sigma-Aldrich, R3501), using a 96-well Scienceware® replicator (Sigma-Aldrich, Z370819-1EA). For each 96-well plate, two technical replicates were plated onto separate KB agar plates and incubated at 28°C for 2 days before CFUs counting. The number of CFUs in replicated plates were averaged. For each plant, bacterial CFUs were normalized to its leaf area (mm^2^).

*Plectosphaerella cucumerina* strain BMM (*Pc*) was cultivated on half-strength Potato Dextrose Agar (BD Difco, BD-213400) for 3.5 weeks in the dark. Spores were resuspended from agar plates in water and filtered through two layers of Miracloth (Merck, 475855-1R) to remove mycelium debris. *Pc* inoculum was adjusted to 10^6^ spores/ml in water, using a Neubauer haemocytometer. The inoculum was applied by pipetting 6-μl droplets (10^6^ spores/ml) onto four fully expanded leaves of similar age. Inoculated plants were covered and kept at 100% humidity. The resistance phenotypes were addressed by measuring the diameter of lesions caused by the pathogen 21 dpi. The lesion diameter of the four infected leaves of each plant were averaged.

*Botrytis cinerea* was cultured for 3.5 weeks at 23°C in Petri dishes with PDA medium supplemented with ground Arabidopsis leaves at 8-h light. Inoculum (2.5 10^6^ spores/ml), and the inoculation procedure was performed as described in Fernández-Santos et al. (2020). The plants were kept at 100% humidity during infection, and the diameter of the lesions caused by the fungus was measured 4 dpi.

### AA Growth Inhibition Test in *Pst*

To rule out a possible toxic effect of AA for *Pst*, a 30-ml culture was set up in the Luria-Bertani (LB) liquid medium from a glycerol stock (*Pst* DC3000) and grown overnight at 28°C with rotation shaking. The culture was stopped at OD_600nm_ = 0.4 (~3.2 × 10^8^ cfu/ml) and diluted 100 times (~3.2 × 10^6^ cfu/ml) to set up an initial preculture. Equal aliquots (50 ml) of the preculture were mixed with the different treatments. Treatments applied were 0-, 0.1- 1-, 10-, and 100-μM Antimycin A or ethanol (Mock). 200 μl aliquots were distributed in different positions of a 96-well standard clear-bottom microtiter plate. A total of 16 replicates per condition and 32 replicates from the non-treatment samples were set up in two different plates and grown at 28°C with rotation shaking. The impact of treatments in the growth of *Pst* was determined by OD_600nm_ measurement, after 24 h, using a Spectra Max ID3 Multimode plate reader.

### Gene Expression Analysis

For gene expression analysis, 2.5-week-old plants were pretreated with 50 μMAA (or Mock) +0.02% tween 20 by spray. Three dat, the seedlings were infected with *Hpa*. Samples for RNA extraction were collected at 0, 2, and 3 dpi (and 3, 5, and 6 dat). Each sample contained around 10 seedlings, and each condition was constituted by three–four different samples (following a randomized block design). RNA was extracted by the method described in Logemann et al. (1987). RNA quality and concentration were assessed by spectrometry, using NanoDrop® (ND-1000) and standardized afterwards to 200 ng/μl. Contaminant DNA was removed by using DNase TURBO DNA-free (AM1907, Invitrogen), and cDNA synthesis was performed, using the Transcriptor First Strand cDNA Synthesis Kit (04897030001, Roche) in a SimpliAmp Thermal Cycler (Applied Biosystems). The relative amount of the transcripts of interest was estimated by q-PCR analysis in a 7,500 Real-Time PCR system (Applied Biosystems), using NZYSpeedy qPCR Green Master Mix (MB22303, Nzytech) and specific primers (Table 1). The CT values obtained in the amplification curves were used to calculate the relative expression of each gene per sample by the method of the reference sample, as described in Rao et al. (2013). The total amount of RNA/sample was corrected, using the expression of the constitutive genes At5g25760 (*UBC*) and At2g28390 (*SAND*) as recommended in Czechowski et al. (2005). Finally, the gene expression values were expressed as relative rates being “1,” the average value of the control, a Mock sample at the beginning of the experiment (Mock pretreatment, Mock challenged, time 0 hpi). Gene expression analysis was performed three times with similar results.

**Table 1:** List of the primers used in the study.

TGTCCCGTTCGCAAACAAGTTC	FW WRKY6 At1g62300 qPCR
CGGCAACGGATGGTTATGGTTTC	RV WRKY6 At1g62300 qPCR
TGGCTTAGATGAGCTCGGTGAAC	FW WRKY29 At4g23550 qPCR
AGCTTGTGAGGATCGTTTGTGTGG	RV WRKY29 At4g23550 qPCR
ATCCCGGCAGTGTTCCAGAATC	FW WRKY53 At4g23810 qPCR
AGAACCTCCTCCATCGGCAAAC	RV WRKY53 At4g23810 qPCR
TGAGCTCGAACCCAAGATGTTCAG	FW WRKY70 At3g56400 qPCR
TGCTCTTGGGAGTTTCTGCGTTG	RV WRKY70 At3g56400 qPCR
CACTACTCCGCAGATCCAACAA	FW S3H At4g10500 qPCR
TCTCCAGTTCAAGACTTTGTCTGC	RV S3H At4g10500 qPCR
TCCATTACGCGGTCACAAAGCC	FW At2g17740 qPCR
TAGGTCGCAACCAGAGCAGATG	RV At2g17740 qPCR
AAGAGTTTCGAGCAGAGGTTGAC	FW FRK1 At2g19190 qPCR
CCAACAAGAGAAGTCAGGTTCGTG	RV FRK1 At2g19190 qPCR
TTCGACATCGCCTTCGACAAGTG	FW MYB15 At3g23250 qPCR
TAGCCGTCGTGGCTTATGAGTG	RV MYB15 At3g23250 qPCR
GTTCACAACCAGGCACGAGG	FW PR1 At2g14610 qPCR
CAAGTCACCGCTACCCCAG	RV PR1 At2g14610 qPCR
TCTCTCTCTCTCTCTCTCGCTCTC	FW UBC At5g25760 qPCR
TGATGCCTGCATCTCTAATTTCCC	RV UBC At5g25760 qPCR
CAAGGCAGGAAATCACCAGGTTG	FW SAND At2g28390 qPCR
CTGTACAGCTGATGCAGACCAG	RV SAND At2g28390 qPCR

### Chromatin Immunoprecipitation (ChIP) Analysis

ChIP analysis was performed in fully expanded leaves from 4-week-old pretreated plants (50 μMAA or Mock + 0.02% tween 20 by spray). Three dat, fully expanded leaves from at least 12 different plants/treatment were included in each of the samples (following a randomized block design). Each condition was constituted by two-four different samples (two replicates were used only in BTH treatments as internal experimental control). Chromatin isolation and analysis were conducted as described in Haring et al. (2007) from 2 g of leaf tissue per sample. Chromatin immunoprecipitation was performed, using EpiQuik Chromatin Immunoprecipitation Kit (P-2002, Epigentek) with the antibody antiH3K4m3 (#07-473 Millipore). Immunoprecipitated samples were quantified by q-PCR analysis in a 7,500 real-time PCR system (Applied Biosystems), using NZYSpeedy qPCR Green Master Mix (MB22303, Nzytech) and specific primers previously reported by Jaskiewicz et al. (2011). Relative levels were calculated by the method of the reference sample, as described in Rao et al. (2013). The total amount of DNA/sample was corrected, using values of input aliquots (non-immunoprecipitated) of each sample. Finally, the values were expressed as relative rates, being “1” the average of the control, Mock. ChIP analyses were performed three times with similar results.

### GUS Staining

GUS histological staining was performed as described in Vellosillo et al. (2007).

## Results

### Mitochondrial Stress Induces Plant Resistance

**Figure 1:**
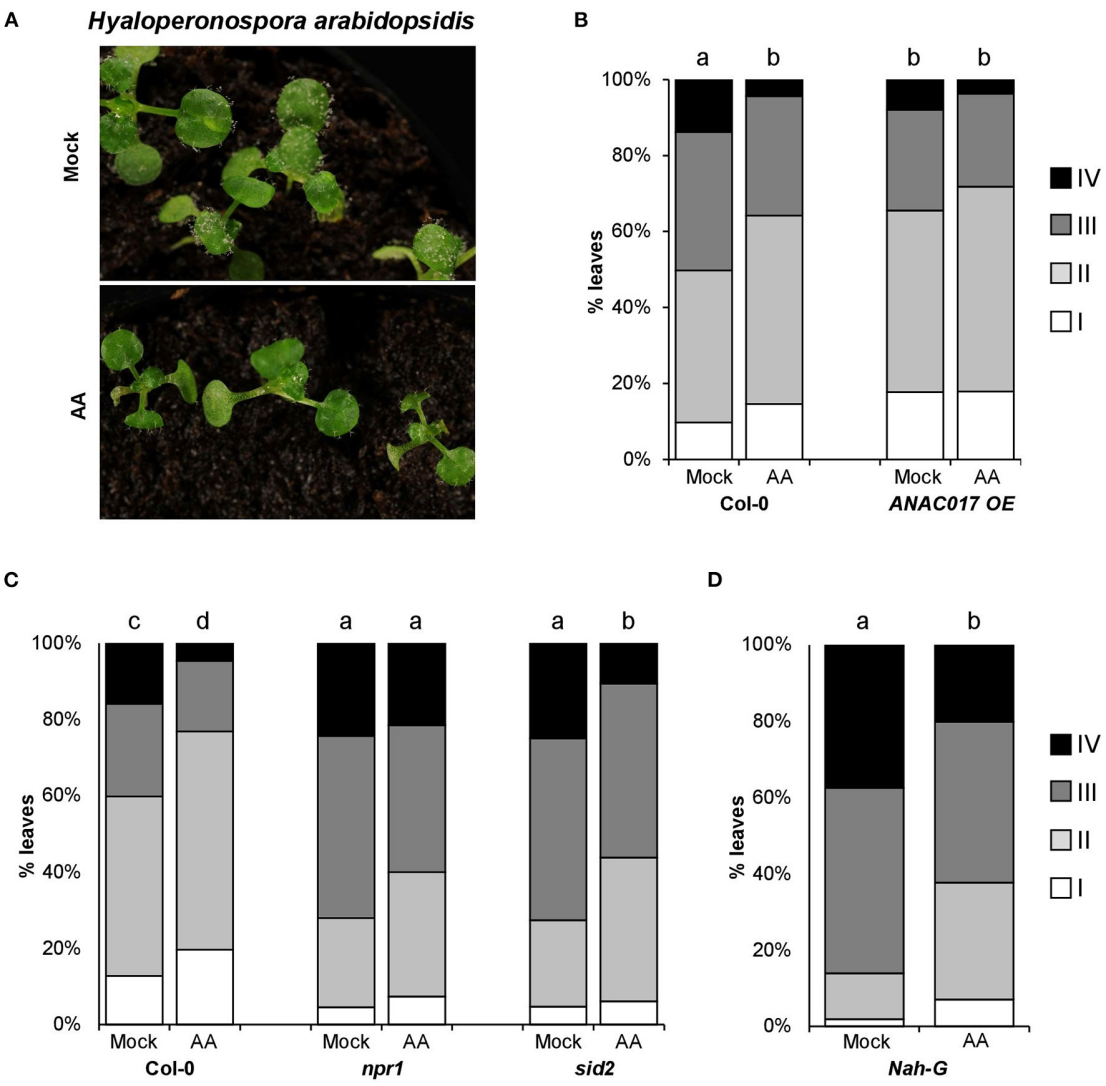
*Hpa* resistance induced by antimycin A treatments. About 2.5-week-old Arabidopsis seedlings were treated with 50-μM antimycin A (AA) or control (Mock) +0.02% tween 20 by spray. Three days after treatment (dat), plants were spray inoculated with *Hpa* at 10^5^ spores/ml. **(A)** Photographs showing AA-IR against *Hpa* 5 days post inoculation (dpi). **(B–D)** AA-induced resistance in different genetic backgrounds. For all genotypes, *Hpa* susceptibility was determined 5 dpi (and 8 dat). The plants were collected in ethanol 96% and trypan blue stained. Infected leaves were analyzed with the help of a stereomicroscope and assigned to one of the four different *Hpa* colonization classes (I: no visible colonization, II: Oomycete growth without sporulation, III: visualization of sporangiophores and asexual sporulation, IV: sexual sporulation). The bar graphs represent the classes distribution in % for the infected leaves analyzed in each genotype. The letters above the bars indicate statistically significant differences by multiple chi-square tests *p* ≤ 0.01. *n* = 150 (in **D**) −590 (in **B** and **C**).

In order to set up an appropriate method to induce mitochondrial stress, we used the reporter line *AOX1a promoter: GUS* fusion to visualize the mitochondrial stress induced by spraying or syringe infiltration with AA (Supplementary Figure 1). *AOX1a* encodes ALTERNATIVE OXIDASE1a, a mitochondrial protein that restores electron transport when ETC is inhibited by antimycin A (AA). As AOX1a is nuclear-encoded and strongly inducible, its expression is considered as a marker for retrograde signaling (Zarkovic et al., 2005; Vanlerberghe, 2013). As previously reported (Umbach et al., 2012), AA treatments at 50 μM triggered mitochondrial stress, activating the retrograde signaling and inducing *AOX1a* gene (Supplementary Figure 1). Next, we analyzed the impact of mitochondrial stress in plant resistance against the biotrophic pathogen *Hyaloperonospora arabidopsidis* (*Hpa*). While the development of *Hpa* conidiophore structures was visible in most of the mock treated plants 5 days after inoculation, it was just perceptible in few of the leaves from the plants treated with AA (Figure 1A). To quantify the observed differences, individually infected leaves were assigned to colonization classes attending to the degree of the pathogen development, and the results represented in stack bar graphs showing the classes distribution in both treatments (Mock *vs*. AA, Figure 1B). As shown in Figure 1A, the induction of mitochondrial stress 3 days before inoculating the plants with the pathogen triggered an evident resistance phenotype. To support the evidence pointing to mitochondrial stress as plant resistance inducer, we included in our analysis a transgenic line overexpressing the protein ANAC017 (Van Aken et al., 2016). ANAC017 is a key factor in the retrograde signaling communication from mitochondria to the nucleus (Clercq et al., 2013; Ng et al., 2013; Broda and Van Aken, 2018; Meng et al., 2019; Broda et al., 2021). The overexpression of this transcriptional factor is well characterized and shows high levels of *AOX1a* (Van Aken et al., 2016; Meng et al., 2019; Broda et al., 2021), indicating a somehow constitutive MS response. In accordance, the plant line overexpressing ANAC017 showed a resistance phenotype against the pathogen in basal conditions (Figure 1B). Moreover, this line was unable to display significant AA-induced resistance, suggesting that the level of resistance triggered by the MS in this line is already close to its maximum.

### Mitochondrial Stress-Induced Resistance (MS-IR) Is SA Independent but NPR1 Dependent

Plant resistance against biotrophic pathogens is extensively mediated by the signaling of the hormone salicylic acid (SA, Bürger and Chory, 2019). By the analysis of plant lines defective in SA production or accumulation {*sid2* mutant [Wildermuth et al. (2001)] and *Nah-G* transgenic lines, respectively}, we observed wild-type levels of MS-IR against *Hpa* in the plant lines analyzed (Figures 1C,D). Accordingly, our results suggest that the induced resistance triggered by mitochondrial stress is largely independent of SA production and accumulation. Conversely, *npr1* mutant plants were impeded in MS-IR against the *Hpa*, pointing to a role of the defense master regulator NPR1 (NONEXPRESSOR OF PR GENE1) in the resistance induced by mitochondrial stress (Figure 1C).

### MS-IR Protects Local, Systemic Tissue, and Transgenerationally

**Figure 2:**
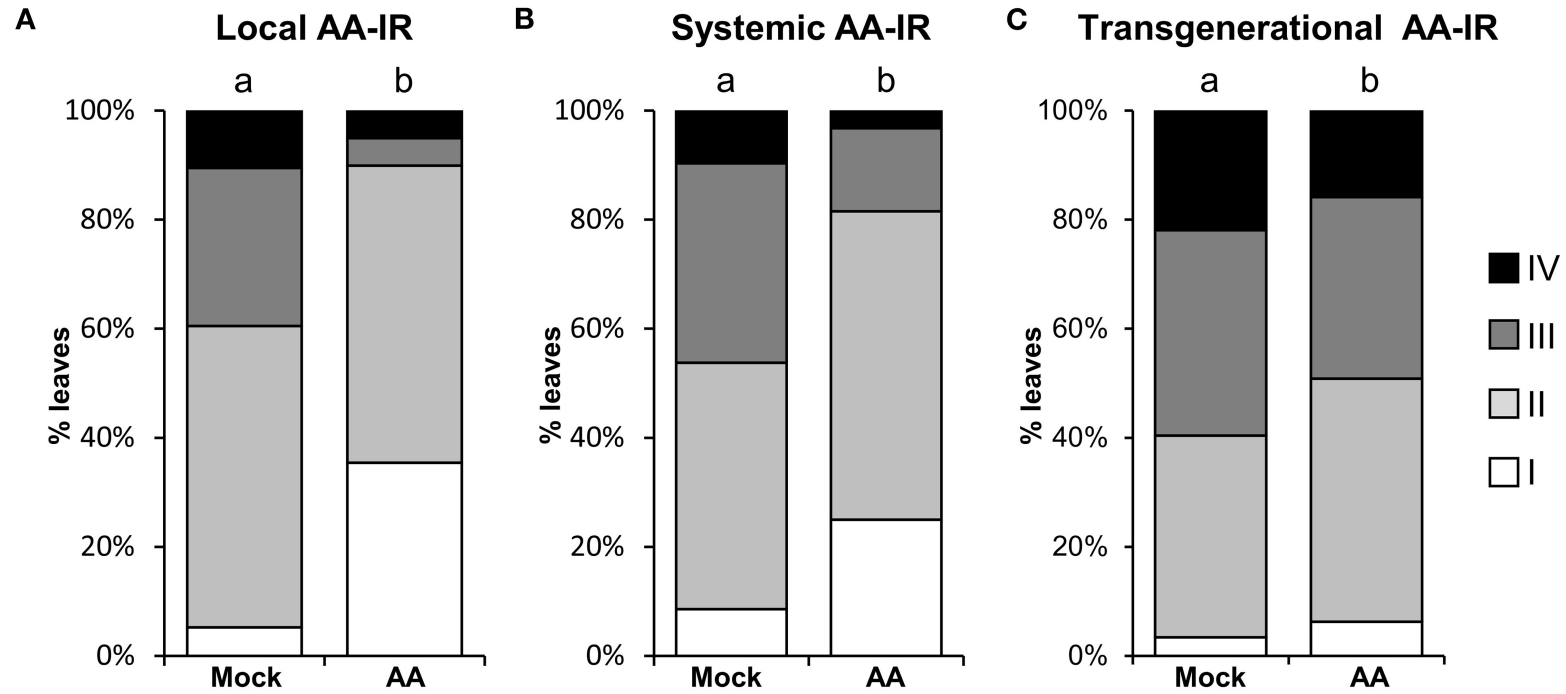
MS-IR is systemic and long-lasting. **(A,B)** 4-week-old plants were locally pretreated with 50 μM AA or Mock by syringe infiltration and infected with *Hpa* at 3 dat. *Hpa* colonization was addressed 5 dpi (8 dat) by trypan blue staining, followed by scoring of the oomycete development with the help of a stereomicroscope (see Figure 1 legend). The letters above the bars indicate statistically significant differences by chi-square tests *p* ≤ 0.01. **(A)** Local AA-IR. *Hpa* colonization was assessed in the pretreated and infected leaves. *n* = 75. **(B)** Systemic AA-IR. *Hpa* colonization was assessed in distal (non-AA/Mock treated leaves from AA/Mock locally treated plants) and infected leaves. *n* = 92. **(C)** Transgenerational IR. *Hpa* colonization was monitored in the direct progeny (F1) of AA or Mock-treated plants. Shown are the results of pooled infected 2.5-week-old seedlings from four lines/treatment. Results from independent lines are shown in Supplementary Figure 2. The letters above the bars indicate statistically significant differences by chi-square tests *p* ≤ 0.01. *n* = 320.

To go deeper into the characterization of mitochondrial stress as a plausible IR stimulus, we first explored whether the MS-IR is specifically displayed in the treated local tissue or a transmission of the defense signal to distal tissues could be involved in the phenotype. Local AA treatments were performed by syringe infiltration in marked leaves of 4-week-old wild-type plants. Three days after treatment (dat), the plants were infected with *Hpa*. As it can be observed in Figure 2, AA treatments elicited resistance both in locally treated (Figure 2A) and systemic (distal) tissues (Figure 2B). The IR elicited by specific agents/stimuli has been recently described to persist for long periods of time, even being transmitted to the next generations of plants (Wilkinson et al., 2019). Considering that, in our previous assays, the inoculations were performed 3 days after the treatment with AA (3 dat), and that the evaluation of pathogen growth was examined at 8 dat, we could conclude that MS-IR was observed at least from 3 to 8 dat. Taking into account the systemic character of the MS-IR, we addressed the transgenerational persistence of the phenotype. The progeny from plants subjected to mitochondrial stress (AA treatments) in the previous generation was statistically more resistant against *Hpa* than the descendants of the control treatments (Figure 2C). This between-generations resistance was less evident than the within-generation resistance; however, we found statistical differences in three out of four lines analyzed (50–100 individuals were analyzed per line, Supplementary Figure 2). Thus, our results suggest that mitochondrial stress is perceived as an IR stimulus, triggering an immunological state (conditioned state), which confers resistance against *Hpa* in locally treated tissues, systemic parts of the plants, and even in following generations.

### MS-IR Mainly Correlates With Priming of Gene Expression

**Figure 3:**
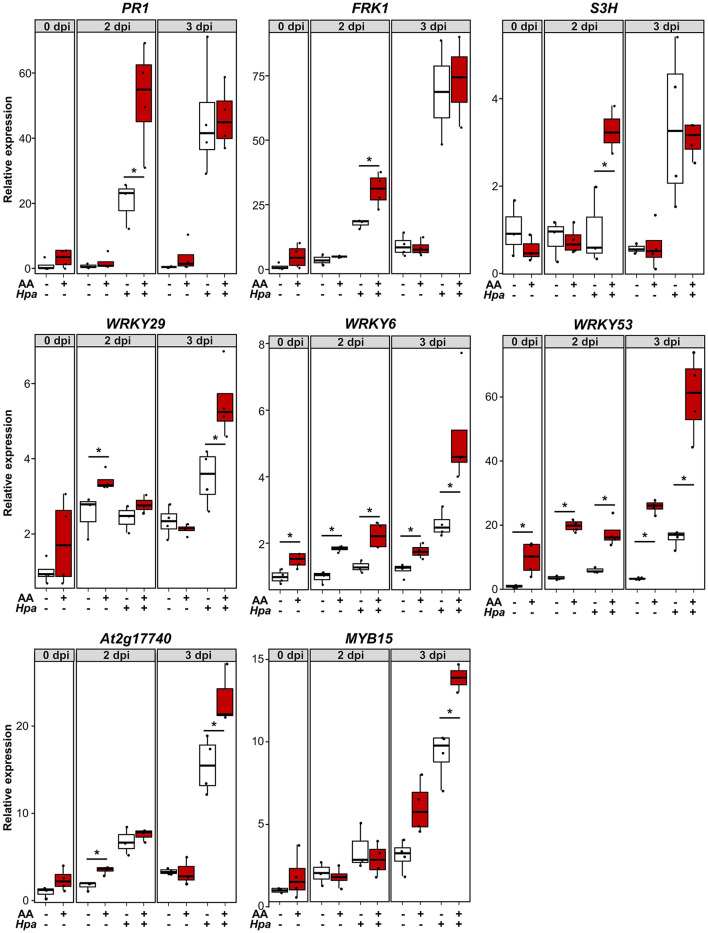
MS-induced resistance is associated with a faster/stronger induction of several defense-related genes. About 2.5-week-old plants were pretreated with 50 μMAA (or Mock) +0.02% tween 20 by spray. Three dat, the seedlings were infected with *Hpa*. Samples for RNA extraction were collected at 0, 2, and 3 dpi. Each sample contained around 10 seedlings. X axis indicates gene expression analyzed by RT-qPCR, using specific primers and relative to Mock pretreated samples at time 0 (pre-inoculation). For each sample, the total amount of RNA was corrected using the expression of the housekeeping genes At5g25760 (*UBC*) and At2g28390 (*SAND*). The boxplot represents the interquartile range (IQR; Q3-Q1: as the distance between the median of the lower half of the data set –Q1- and highest half –Q3-, including the central 50% of the data). The horizontal line inside the boxes represents the sample median. Whiskers are drawn ± the last datapoint within 1.5 times the IQR. Replication units are shown as overlaying jittered-dot-plots. Asterisks label statistically significant differences between AA and Mock pretreated samples (*p* < 0.05, Student's *T*-test), *n* = 3–4.

Behind IR phenotypes, there is usually a combination of direct (induction of specific mechanisms as a response to the IR agent) and primed defense responses (when the main induction of defense responses only occurs in plants pretreated with the IR agent in the presence of a pathogenic challenge). The weight of each factor determines the relative importance of the underlying defense mechanisms in the resistance phenotype (De Kesel et al., 2021). To explore the contribution of direct and primed defense responses, we assessed the expression dynamics of different defense-related genes during the course of infection with *Hpa* (as a challenge). We compared plants pretreated with AA as an MS-IR agent *vs*. Mock pretreated plants. Three dat, we infected the seedlings with the pathogen and collected tissue for gene expression analysis at days 2 and 3 post inoculation. The selected times were consistent with previous reports addressing priming analyses (López Sánchez et al., 2016). In Figure 3, the different gene expression patterns are presented. Some of the early induction-analyzed genes (*PR1, FRK1*, and *S3H*) showed a typical pattern of primed induction of gene expression. For this set of genes, statistically significant differences between AA and control pretreated plants were visible after pathogen inoculation, but not in response to AA (time 0) or Mock treatments. Thus, these genes are expressed faster when pretreated with AA and subjected to the challenge (*Hpa*), and, as a reflection, the differences appear at short-time points of infection, i.e., 2 days postinoculation (dpi). We identified another set of defense-related genes whose expression is at least partially induced by the AA pretreatment (differences at time 0) and for which the sensitization caused by the mitochondrial stress seems to increase their inducibility, responding more efficiently even to Mock challenge treatments (pretreated with AA but Mock infected). It is the case of *WRKY29, WRKY6, WRKY53*, and At2g17740. This could reflect a partial contribution of direct defenses to the MS-IR phenotype. However, in all cases, the greatest differences were found between AA and Mock pretreated samples at long time points (3 dpi) in infected samples, suggesting a stronger contribution of priming mechanisms to the MS-IR phenotype. Finally, for the *MYB15* gene, we observed priming of a gene expression pattern, which appears at the latest time point (3 dpi).

### MS-IR Associates With Epigenetic Changes

**Figure 4:**
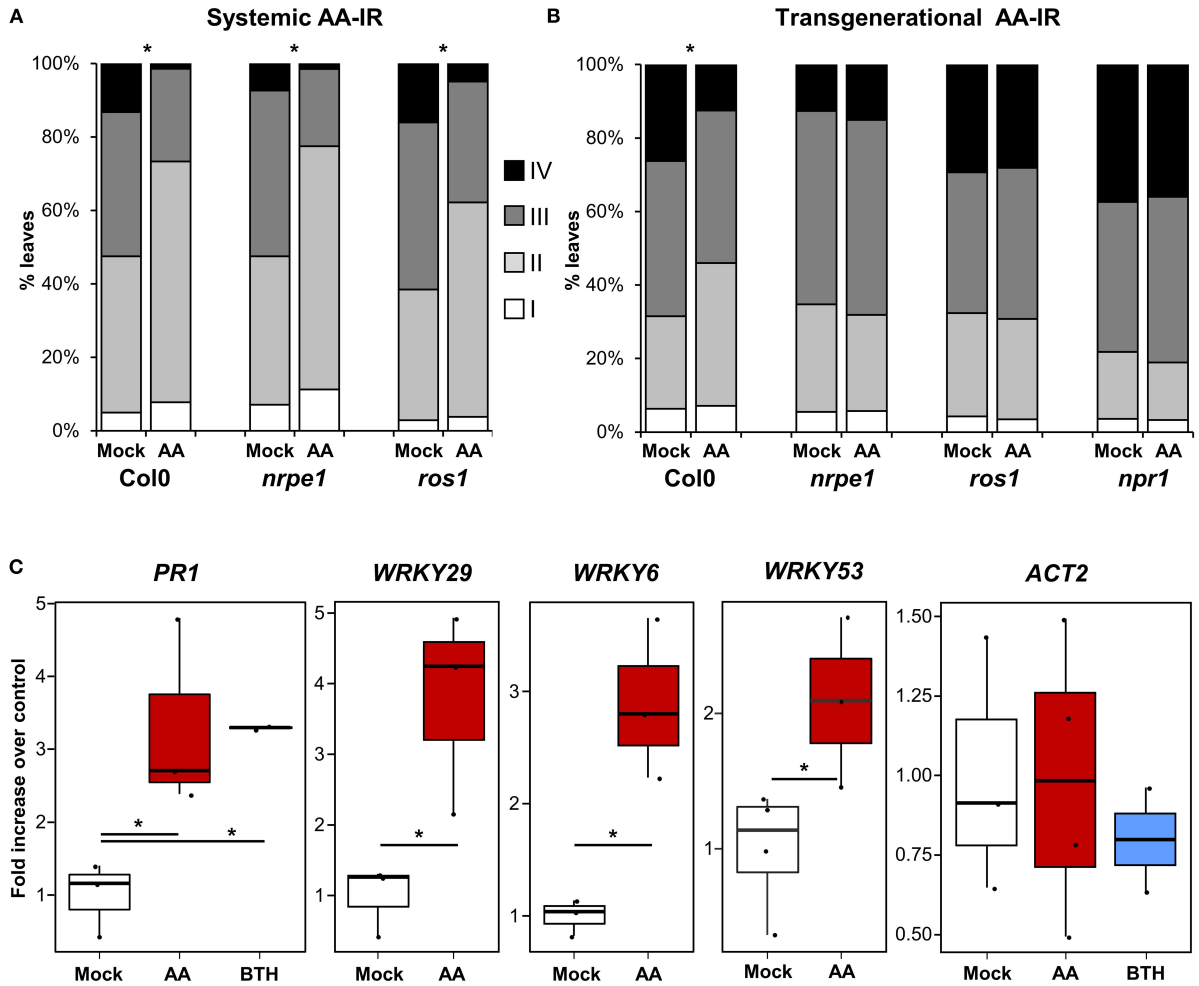
MS-IR associates with epigenetic changes. **(A,B)** 2.5-week-old Arabidopsis seedlings were spray inoculated with *Hpa* at 10^5^ spores/ml. *Hpa* growth was determined 5 dpi. Trypan blue-stained leaves were scored and assigned to one of the four different *Hpa* colonization classes (Figure 1 legend). Bar graphs represent the classes distribution in % for the infected leaves analyzed in each genotype/treatment. **(A)** AA systemic IR in DNA (de)methylation mutants. Arabidopsis seedlings were treated with 50-μM antimycin A (AA) or control (Mock) +0.02% tween 20 by spray. Plants were spray inoculated with *Hpa* at 3 dat. Asterisks label statistically significant differences between Mock and AA pretreated samples (chi-square test *p* < 0.01), *n* = 200–400. **(B)** AA t-IR in DNA (de)methylation mutants. *Hpa* colonization was monitored in the direct progeny (F1) of AA or Mock-treated plants. Shown are the results of pooled-infected 2.5-week-old seedlings from four lines/treatment. Results from independent lines are shown in Supplementary Figure 3. Asterisks label statistically significant differences between Mock and AA pretreated progenitors (chi-square tests *p* < 0.01). *n* = 850–110. **(C)** Chromatin changes at the promoter region of defense-related genes. Chromatin immunoprecipitation (ChIP) analysis was performed in Mock/AA/BTH pretreated plants (no challenged), using specific antibodies against the positive mark H3K4me3. Samples are pools of at least 12 plants (fully expanded leaves). Replicates are independent samples from a random block experimental design (total population analyzed 24–48 plants per treatment). The boxplots represent the interquartile range (IQR; Q3–Q1, see Figure 3 legend for further details). The horizontal line inside the box represents the sample median. Whiskers are drawn ± the last datapoint within 1.5 times the IQR. All replication units are shown as overlaying jittered-dot-plots. Asterisks label statistically significant differences between Mock and AA/BTH pretreated samples (*p* < 0.05, Student's *T*-test), *n* = 2–4 (two replicates were only used in BTH treatments as an internal experimental control).

Among the molecular mechanisms underlying induced resistance, epigenetics seems to play an important role in priming of defense responses (Alonso et al., 2019). Changes in DNA methylation occur in response to pathogen attack (Dowen et al., 2012) and are required for transmission of the primed immunological state to the next generations of plants in transgenerational IR (t-IR) experiments (Luna and Ton, 2012; López Sánchez et al., 2016; Stassen et al., 2018). In previous studies and by the use of mutants defective in DNA (de)methylation processes, we observed the requirement of intact DNA methylation machinery, not in within-generation IR phenotypes, but in the transmission of those phenotypes transgenerationally (López et al., 2011; López Sánchez et al., 2016). We then analyzed the ability of the mutants *nrpe1* and *ros1* (defective in DNA methylation and demethylation processes, respectively) in triggering MS-IR both within generation and transgenerationally. The DNA (de)methylation mutants displayed an unaltered MS-IR at 3 dat (Figure 4A). However, similar to the case of pathogen-induced resistance, those mutant plants did not show t-IR elicited by mitochondrial stress (Figure 4B; Supplementary Figure 3), demonstrating the requirement of DNA methylation changes for the stability of the phenotype. Despite the close relationship between DNA methylation changes and t-IR (Luna and Ton, 2012; Stassen et al., 2018), targets of the DNA methylation machinery mediating this t-IR are still elusive. However, specific chromatin changes, such as an increment in the trimethylation of H3K4 (H3K4me3), have been reported after pathogenic stimulus or application of IR agents (Jaskiewicz et al., 2011; López et al., 2011), suggesting that these inductive stimuli can fingerprint defense genes with open chromatin marks, facilitating their subsequent induction during the challenge. Moreover, this type of epigenetic marks also appears in transgenerationally induced resistance (t-IR) lines and are constitutively present in DNA methylation mutant plants (López et al., 2011; Luna and Ton, 2012; López Sánchez et al., 2016). Considering the systemic and transgenerational character of MS-IR and its association with priming of defense gene expression (some of which have been previously described as targets of the epigenetic machinery), we decided to analyze the deposition of the positive mark H3K4me3 in response to mitochondrial stress. As an internal positive control for the experiment, we used parallel treatments with the IR agent BTH (Jaskiewicz et al., 2011), and, as a negative control, we tested the deposition of this mark in an unrelated gene (*ACT2*). As shown in Figure 4C, by ChIP analysis, an increment of H3K4me3 at the promoter region of *PR1* gene was detected in BTH primed samples, whereas it was absent in *ACT2*, confirming the appropriate setups of the technique. In plants pretreated with AA (elicited with mitochondrial stress), we observed an increment of this epigenetic mark at the promoter regions of the genes *PR1, WRKY29, WRKY9*, and *WRKY53*, further supporting the role of epigenetics in the MS-IR (Figure 4C).

### Mitochondrial Stress Induces Broad Spectrum Resistance

**Figure 5:**
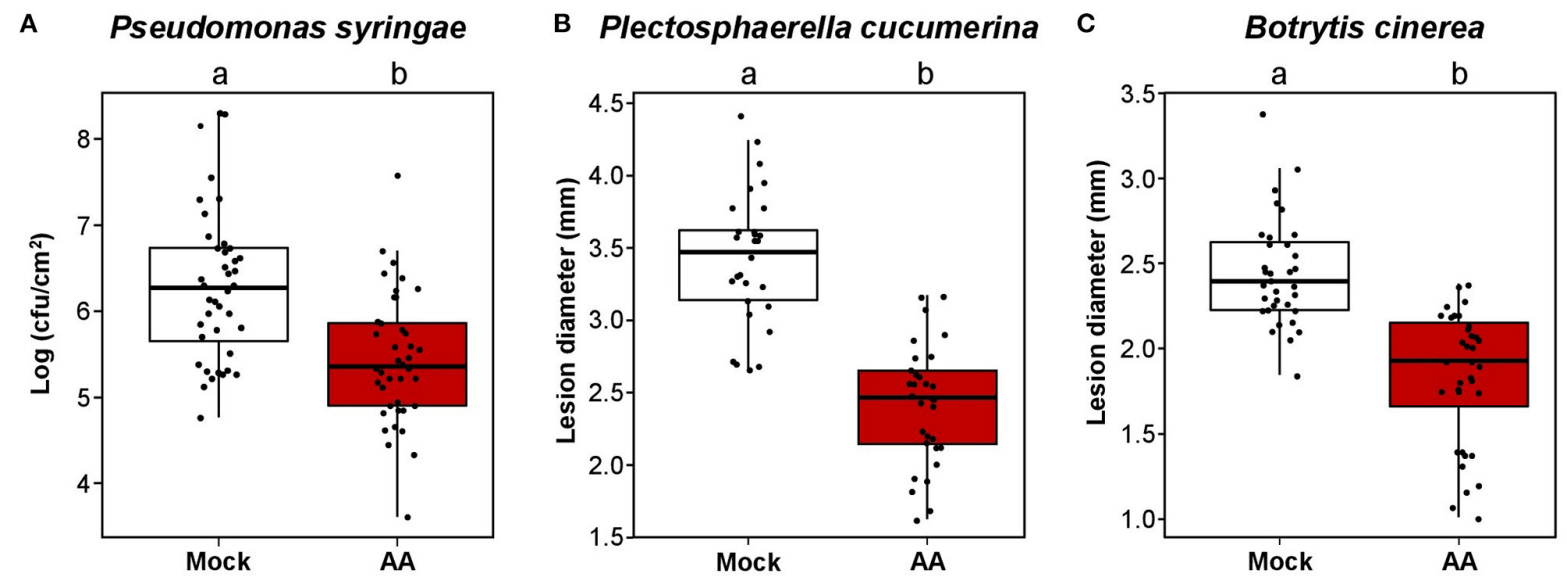
Mitochondrial stress induces broad spectrum resistance. Boxplots represent the interquartile range (IQR; Q3–Q1). The median value is shown as the horizontal line inside the boxes. Whiskers are drawn ± the last datapoint within 1.5 times the IQR. Overlaying jittered-dot-plots represent all replication units. Letters above the boxplots indicate statistical differences, *p* ≤ 0.05, Student's *T*-test. In all experiments, 4.5-week-old plants were treated with 50 μM AA or Mock. Pathogen inoculation was performed 3 dat. **(A)** AA-IR against *Pseudomonas syringae* pv *tomato* DC3000 (*Pst*). The plants were spray inoculated with *Pst* at OD:0.06. Three dpi (6 dat), the growing of the bacteria was assessed by direct counting of individual colonies from leave tissue extractions and serial dilutions followed by plating in KB medium plates. Replication unit = plant (three leaves from the same plant were collected per sample). *n* = 40–41. **(B)** AA-IR against *Plectosphaerella cucumerina* (*Pc*). The plants were drop-inoculated with the solution of a spore of *Pc* at 10^6^ spores/ml. Twenty-one dpi (24 dat), the growing of the fungus was assessed by measuring the lesion diameter from infected leaves. Replication unit = plant (the lesion diameter of the four infected leaves per plant was averaged in each replication unit). *n* = 28–30. **(C)** AA-IR against *Botrytis cinerea* (*Bc*). The plants were drop-inoculated with the solution of a spore of *Bc* at 2.5 10^6^ spores/ml. Four dpi (7 dat), the growing of the fungus was assessed by measuring the lesion diameter from infected leaves. Replication unit = plant (the lesion diameter of the four infected leaves per plant was averaged in each replication unit). *n* = 31.

To address the resistance spectrum of MS-IR, we subjected adult plants pretreated with AA (or Mock) against a variety of pathogens. First, we performed inoculations with the model bacterial pathogen *Pseudomonas syringae* pv. *tomato* (Xin and He, 2013), using the virulent strain DC3000 (*Pst* DC3000). *Pst* is considered to be a hemi-biotrophic pathogen, phylogenetically unrelated to *Hpa*, but both are mainly resisted by the salicylic acid hormonal pathway (Bürger and Chory, 2019). As it is shown in Figure 5A, plants pretreated with AA displayed evident resistance against this pathogen. At this point, although we previously demonstrated the mitochondrial stress effect on the plant defense mechanisms, we addressed the plausible direct inhibition of the pathogens by AA. However, in toxicity assays with *Pst* cultures under increasing concentrations of AA, no reduction in the pathogen growth was observed (Supplementary Figure 4). To complement our analysis, we confronted plants pretreated with AA (or Mock) to two different necrotrophic fungi, *Plectosphaerella cucumerina* (*Pc*) and *Botrytis cinerea* (*Bc*). Both pathogens are considered to have different lifestyles when compared with biotrophic pathogens and mainly resisted by the jasmonic acid (JA) hormonal pathway (Bürger and Chory, 2019). As shown in Figures 5B,C, plants pretreated with AA were more resistant to both pathogens, indicating that the mitochondrial stress induced by the AA treatments confers protection against a broad variety of pathogens.

## Discussion

Plant-induced resistance has become one of the most intriguing and attractive research fields in the past decades. Defined as the reduced susceptibility caused by the previous perception of a pathogen or a pathogen-related signal (Mauch-Mani et al., 2017; De Kesel et al., 2021), the potential of induced resistance in the design of novel approaches to control plant pests and diseases is undoubtedly appealing (Worrall et al., 2012; Ramírez-Carrasco et al., 2017; Pétriacq et al., 2018). There have been important efforts focused on understanding such a memory effect, which confers resistance in the absence of new resistance genes (Hammerschmidt, 1999). However, the events taking place after the pathogen perception and leading to the different types of IR phenotypes are mostly unknown. Within this framework, here, we unveiled and characterized a role of mitochondrial stress in IR processes.

We first observed a clear mitochondrial stress-induced resistance (MS-IR) phenotype against the biotrophic pathogen *Hpa* and revealed the action of AA as an IR agent. Investigating the main aspects of MS-IR, we observed this phenotype locally, systemically, and even in the next generation of plants. The molecular mechanisms underlying the IR phenotypes are consensually grouped in two main components (Wilkinson et al., 2019). On the one hand, after the exposure to an eliciting agent, the plant-inducible defenses can remain upregulated, or even accumulate in an inactive form. This is considered a direct or constitutive induction of defenses. On the other hand, the eliciting agent can set up a primed state of plant-inducible defenses. In primed plants, defense levels remain indistinguishable from naïve plants (which did not face the elicitors), but they are expressed more efficiently when the elicited plants face the challenge. Latest studies in the field have considered the relative contribution of direct and primed defenses as a characteristic feature of specific types of IR (De Kesel et al., 2021). In the case of mitochondrial stress as an eliciting agent, our results mostly show a more efficient induction of defense genes during infection rather than their constitutive activation, pointing to a major contribution of priming mechanisms to MS-IR. Even when the role of mitochondrial stress in priming processes was unexplored until date, these observations are in accordance with previous studies on mitochondrial tricarboxylic acid (TCA) cycle. For example, BABA treatments used to induce resistance increase the primary metabolites of the TCA cycle (Pastor et al., 2014), and specific TCA intermediaries, such as citrate and fumarate, have been demonstrated to play a role as IR agents against *Pst* (Balmer et al., 2018). Therefore, it is possible that mitochondrial stress could trigger a disruption of the TCA cycle, promoting the accumulation of some of those intermediaries mediating priming of defenses and the MS-IR phenotype.

Epigenetic mechanisms have been recently involved in priming processes, offering a molecular explanation to the phenotypic plasticity conferred by priming, in the absence of genetic changes (Alonso et al., 2019). Our results suggest that mitochondrial stress could lead to a direct modification of the chromatin through histone changes, labeling the promoter regions of the primed defense genes as open chromatin and facilitating its subsequent induction in the presence of the challenge (priming of gene expression). In addition, mutants defective in the DNA (de)methylation machinery were unable to display transgenerational MS-IR, which proves that an intact DNA (de)methylation epigenetic machinery is required for the transgenerational character of MS-IR. Thus, our results suggest that certain epigenetic changes would be part of retrograde signaling, communicating the mitochondria with the nucleus. These observations could serve as a starting point to search for such communicators generated from stressed mitochondria, with the ability to modify the epigenome. A plausible hypothesis could be an impact on folate synthesis, which is consistent with the metabolic changes derived from mitochondrial stress. The major methyl donor for DNA and histone modifications is S-Adenosylmethionine (SAM), whose synthesis is controlled by the 1C metabolism pathway (folate pathway), which partially takes place at the mitochondria (Gorelova et al., 2017). Thus, it is also possible that mitochondrial stress disrupts folate synthesis, altering the availability of methyl groups for the epigenetic machinery. Indeed, folate availability has been related with both epigenetic changes and priming processes in response to biotic stress (Smith and Butler, 2018; González and Vera, 2019). Other molecules-communicating mitochondria and the epigenome are PARP/PARG [Poly (ADP-ribose) Polymerases/Glycohydrolases] enzymes. PARP/PARG are affected by the mitochondrial levels of NAD+/ATP and control the polyADP-ribosylation of chromatin elements, such as H1 histone variants, contributing to the local relaxation of the chromatin (Rissel and Peiter, 2019). PARP/PARG enzymes have been extensively related with plant defense (Adams-Philips et al., 2010; Feng et al., 2015; Song et al., 2015), appearing as additional candidates to control the mitochondrial-epigenome communication. On the other hand, the protein NPR1 is considered a master regulator of responses against pathogens (Withers and Dong 2016), playing a key role in priming both intra and transgenerationally (Luna et al., 2012, 2014; Withers and Dong, 2016). After pathogen perception, a mitochondria-mediated redox cascade is triggered (Mou et al., 2003; Jin et al., 2018), leading to the reduction of NPR1 by S-nitrosylation (Tada et al., 2008). This reduction is essential to monomerize NPR1 and its translocation to the nucleus, where it acts in priming by remodeling the chromatin structure of defense-related genes (Jin et al., 2018). In our analysis, the mutant *npr1* is impeded in AA-induced resistance both within and between generations. This could indicate that the redox changes that favor NPR1 monomerization and its translocation to the nucleus are triggered by mitochondrial stress and required for the resistance phenotype. Considering the mitochondria-epigenome link presented in this work, these different hypotheses would need to be addressed in future research, specifically focused on discovering the mitochondria-epigenome link.

The fact that mitochondrial stress induces resistance against both biotrophic and necrotrophic pathogens is not unique, yet it is intriguing. Most parts of the previously studied IR processes tend to protect plants against pathogens with lifestyles similar to the one eliciting the response. This is especially evident in natural t-IR (López Sánchez et al., 2021), and it has been attributed to the cross talk between the different hormonal pathways. While SA plays a key role in defense processes against biotrophic pathogens, JA is considered the main hormonal pathway in resisting attacks of necrotrophic ones (Bürger and Chory, 2019), and the antagonism between both hormonal pathways is well established (Koornneef and Pieterse, 2008; Pieterse et al., 2012). Here, we report the broad-spectrum character of MS-IR and its independence from the SA hormonal pathway but dependence on NPR1. This NPR1 dependence is relatively common to other types of IR even when they seem to rely on different hormonal pathways. For example, the most studied form of IR is systemic acquired resistance (SAR, Ross, 1961; Durrant and Dong, 2004), which is triggered by a localized infection with necrotizing pathogens and has been demonstrated to require both the SA hormonal pathway and NPR1. Nonetheless, induced-systemic resistance (ISR) (Loon et al., 1998; Pieterse et al., 2014) is triggered by beneficial microbes in the rhizosphere; it is NPR1 and JA dependent but SA independent. In addition, IR elicited by exogenous applications of BABA has been described to be, at least, partially dependent on NPR1, especially for the long-lasting phenotypes (Slaughter et al., 2012; Luna et al., 2014). Therefore, our results support the previously suggested role of NPR1 as a link molecule between different types of IR. In fact, NPR1 monomeric forms migrating to the nucleus contribute to the induced resistance against biotrophic pathogens, whereas it is required in its cytoplasmatic location for the induced resistance against necrotrophic pathogens (Kinkema et al., 2000; Ramírez et al., 2010). Additionally, part of the BABA-IR, which is SA independent, is mediated by the reinforcement of the external barriers such as the increment in callose deposition. At this respect, important roles of mitochondrial changes in processes like callose deposition have also been reported (Vellosillo et al., 2007, 2013), being a plausible role of these barriers in some of the MS-IR phenotypes. Evidencing the limitations of our study whether the MS-IR against necrotrophs and biotrophs shares molecular mechanisms or follows independent intracellular pathways still needs to be elucidated. Future research should also address how the signal against pathogens with different lifestyles diverges to prime defenses and the role of mitochondria in such cellular decisions. In addition, analyzing the role of mitochondrial stress in other IR processes, such as SAR, ISR, or BABA-IR, would provide valuable knowledge about how plants integrate external stimuli and trigger IR with different specificities. Complementing those analyses, the role of mitochondrial stress in the resistance against abiotic stresses is another interesting field of research.

In summary, in this study, we took the first steps in the identification of intracellular elements mediating IR, positioning mitochondria as a plausible integrating platform for external and internal signals leading into IR phenotypes by epigenetic-lasting changes. These results could offer a key understanding of the cellular link between IR processes and naturally occurring epigenetic changes, stepping forward in the fundamental knowledge of epigenetics and plant defense, as well as opening doors to include controlled forms of IR in the future and more sustainable crop protection strategies.

## Data Availability Statement

The original contributions presented in the study are included in the article/Supplementary Material, further inquiries can be directed to the corresponding authors.

## Author Contributions

AL and CC conceived the project, designed, and supervised the experiments AL, SH, BL, and TC performed bioassays AL, YI, and CC wrote the manuscript. All the authors reviewed and approved the final manuscript. All the authors declare no competing interests.

## Funding

The work was supported by the MSCA fellowship (No. 309944 EPILIPIN) to AL, BIO2015-68130-R (Ministry of Economy and Competitiveness/European Regional Development Fund) and RTI2018- 097102-B-100 (Ministry of Science, Innovation and Universities) Grants to CC. PhD scholarship to BL was supported by BES-2016-076425 (Severo Ochoa Centers of Excellence Program, Ministry of Economy and Competitiveness), and postdoctoral contract to AL was supported by Grant 20172SEV647 (Severo Ochoa Centers of Excellence Program, Ministry of Science, Innovation and Universities).

## Conflict of Interest

The authors declare that the research was conducted in the absence of any commercial or financial relationships that could be construed as a potential conflict of interest.

## Publisher's Note

All claims expressed in this article are solely those of the authors and do not necessarily represent those of their affiliated organizations, or those of the publisher, the editors and the reviewers. Any product that may be evaluated in this article, or claim that may be made by its manufacturer, is not guaranteed or endorsed by the publisher.
