# Supplementary material for: Mitochondrial Stress Induces Plant Resistance Through Chromatin Changes
Source: Front Plant Sci. 2021 Sep 22;12:704964. doi: 10.3389/fpls.2021.704964 (PMC8493246; doi:10.3389/fpls.2021.704964)
Supplement: Supplementary file 1 [file Data_Sheet_1.docx]

Supplementary Material

# Supplementary Figures and Tables

**Supplementary Figure 1.** ***AOX1a* induced expression by AA treatments.** Transgenic plants carrying the construct *AOX1a promoter:GUS* were used to validate the induction of mitochondrial stress in plants treated with AA by syringe infiltration or spray inoculation. 4.5-Week-old plants were treated with 50 µM AA or Mock. 24 hours after treatment (hat), treated leaves were collected and analysed for GUS induction by histochemical analysis.

**Supplementary Figure 2.** **AA t-IR in individual F1 populations against *Hpa*.** Plants in the parental generation (4.5-weeks-old) were exposed 6 times to 50 μM AA or mock over a 3-week period (each 3-4 days). After the treatments, plants were transferred to long-day conditions to trigger flowering and set seed. The seeds of each progeny were collected and analysed as independent lines (from 1 to 4 per each treatment in the parental generation). 2.5-week-old seedlings, F1 progenies from the 8 different lines, were infected with *Hpa* by spraying with a 10^5^ spores/ml solution. 5 dpi, infected seedlings were collected in ethanol 96%, trypan blue stained, visualized with the help of a stereo microscope and assigned to one of the 4 different *Hpa* colonization classes (Figure 1 legend). The bar graphs represent the classes distribution in % for the infected leaves analysed in each line. Asterisk above the bars indicate statistically significant differences by chi-square tests for each F1 AA line *vs* the pooled distribution of F1 Mock population. p ≤ 0.01. n=50 – 100/line.

**Supplementary Figure 3. AA t-IR in individual F1 populations against *Hpa* in different mutant plants.** Plants in the parental generation (4.5-weeks-old) were exposed 6 times to 50 μM AA or mock over a 3-week period (each 3-4 days). Further details in Supplementary Figure 2 legend. 2.5-week-old seedlings, from F1 progenies, were infected with *Hpa* by spraying with a 10^5^ spores/ml solution. 5 dpi, infected seedlings were collected, trypan blue stained, visualized with the help of a stereo microscope and assigned to one of the four different *Hpa* colonization classes (Figure 1 legend). The bar graphs represent the classes distribution in % for the infected leaves analysed in each line. Asterisk above the bars indicate statistically significant differences by chi-square tests for each F1 AA line *vs* the pooled distribution of F1 Mock population (of each genotype). p < 0.01. n=150 – 350/line.

**Supplementary Figure 4. AA toxicity test in *Pst* based in bacterial growth inhibition.** Boxplots represent the Interquartile Range (IQR; Q3-Q1). The median value is shown as the horizontal line inside the boxes. Whiskers are drawn ± the last datapoint within 1.5 times the IQR. Overlaying jittered-dot-plots represent all replication units. Bacterial culture standardized at 3x10^6^ cfu/ml (by OD at 0.4 and 1 in 100 dilution), was exposed to increasing concentrations of AA or mock in the media. Bacterial growth was analysed by optical density (λ= 600nm). Y axis represents the OD measurements for the different samples 24 hours after the treatments. Replication unit = independent cultures. n= 14-16.

**Table 1. List of the primers used in the study.**

| TGTCCCGTTCGCAAACAAGTTC | FW WRKY6 At1g62300 qPCR | |
| --- | --- | --- |
| CGGCAACGGATGGTTATGGTTTC | RV WRKY6 At1g62300 qPCR | |
| TGGCTTAGATGAGCTCGGTGAAC | FW WRKY29 At4g23550 qPCR | |
| AGCTTGTGAGGATCGTTTGTGTGG | RV WRKY29 At4g23550 qPCR | |
| ATCCCGGCAGTGTTCCAGAATC | FW WRKY53 At4g23810 qPCR | |
| AGAACCTCCTCCATCGGCAAAC | RV WRKY53 At4g23810 qPCR | |
| TGAGCTCGAACCCAAGATGTTCAG | FW WRKY70 At3g56400 qPCR | |
| TGCTCTTGGGAGTTTCTGCGTTG | RV WRKY70 At3g56400 qPCR | |
| CACTACTCCGCAGATCCAACAA | FW S3H At4g10500 qPCR |  |
| TCTCCAGTTCAAGACTTTGTCTGC | RV S3H At4g10500 qPCR |  |
| TCCATTACGCGGTCACAAAGCC | FW At2g17740 qPCR |  |
| TAGGTCGCAACCAGAGCAGATG | RV At2g17740 qPCR |  |
| AAGAGTTTCGAGCAGAGGTTGAC | FW FRK1 At2g19190 qPCR |  |
| CCAACAAGAGAAGTCAGGTTCGTG | RV FRK1 At2g19190 qPCR |  |
| TTCGACATCGCCTTCGACAAGTG | FW MYB15 At3g23250 qPCR |  |
| TAGCCGTCGTGGCTTATGAGTG | RV MYB15 At3g23250 qPCR |  |
| GTTCACAACCAGGCACGAGG | FW PR1 At2g14610 qPCR |  |
| CAAGTCACCGCTACCCCAG | RV PR1 At2g14610 qPCR |  |
| TCTCTCTCTCTCTCTCTCGCTCTC | FW UBC At5g25760 qPCR |  |
| TGATGCCTGCATCTCTAATTTCCC | RV UBC At5g25760 qPCR |  |
| CAAGGCAGGAAATCACCAGGTTG | FW SAND At2g28390 qPCR |  |
| CTGTACAGCTGATGCAGACCAG | RV SAND At2g28390 qPCR |  |
